# Endothelin receptor B antagonists decrease glioma cell viability independently of their cognate receptor

**DOI:** 10.1186/1471-2407-8-354

**Published:** 2008-11-28

**Authors:** Jennifer P Montgomery, Paul H Patterson

**Affiliations:** 1 California Institute of Technology, 1200 E. California Blvd, MC 216-76, Pasadena, CA 91125, USA

## Abstract

**Background:**

Endothelin receptor antagonists inhibit the progression of many cancers, but research into their influence on glioma has been limited.

**Methods:**

We treated glioma cell lines, LN-229 and SW1088, and melanoma cell lines, A375 and WM35, with two endothelin receptor type B (ETRB)-specific antagonists, A-192621 and BQ788, and quantified viable cells by the capacity of their intracellular esterases to convert non-fluorescent calcein AM into green-fluorescent calcein. We assessed cell proliferation by labeling cells with carboxyfluorescein diacetate succinimidyl ester and quantifying the fluorescence by FACS analysis. We also examined the cell cycle status using BrdU/propidium iodide double staining and FACS analysis. We evaluated changes in gene expression by microarray analysis following treatment with A-192621 in glioma cells. We examined the role of ETRB by reducing its expression level using small interfering RNA (siRNA).

**Results:**

We report that two ETRB-specific antagonists, A-192621 and BQ788, reduce the number of viable cells in two glioma cell lines in a dose- and time-dependent manner. We describe similar results for two melanoma cell lines. The more potent of the two antagonists, A-192621, decreases the mean number of cell divisions at least in part by inducing a G2/M arrest and apoptosis. Microarray analysis of the effects of A-192621 treatment reveals up-regulation of several DNA damage-inducible genes. These results were confirmed by real-time RT-PCR. Importantly, reducing expression of ETRB with siRNAs does not abrogate the effects of either A-192621 or BQ788 in glioma or melanoma cells. Furthermore, BQ123, an endothelin receptor type A (ETRA)-specific antagonist, has no effect on cell viability in any of these cell lines, indicating that the ETRB-independent effects on cell viability exhibited by A-192621 and BQ788 are not a result of ETRA inhibition.

**Conclusion:**

While ETRB antagonists reduce the viability of glioma cells *in vitro*, it appears unlikely that this effect is mediated by ETRB inhibition or cross-reaction with ETRA. Instead, we present evidence that A-192621 affects glioma and melanoma viability by activating stress/DNA damage response pathways, which leads to cell cycle arrest and apoptosis. This is the first evidence linking ETRB antagonist treatment to enhanced expression of DNA damage-inducible genes.

## Background

The endothelin (ET) family includes three 21-amino acid peptides, ET-1, ET-2 and ET-3, which bind to two G-protein-coupled receptors, endothelin receptor type A (ETRA) and endothelin receptor type B (ETRB). The ETRA binds ET-1 and ET-2 with equal preference over ET-3, while ETRB binds all three isoforms with equal affinity [1]. The ET axis is believed to play a role in various malignancies including ovarian, prostate, cervical and breast carcinomas, melanoma and central nervous system tumors [2]. The influence of the ET family on cancer is multifactorial: ET-1 induces proliferation [3-7], suppresses apoptosis [8], enhances angiogenesis [9,10] and promotes invasion [11-13].

Components of the ET system have been found in many glioma tumor specimens and cell lines, and ET expression positively correlates with the degree of malignancy [14-17]. Two studies demonstrated ETRA expression in the neovasculature of glioblastoma tumors, while ETRB was localized to the tumor cells [18,19]. Inhibitors of ET converting enzyme 1, which converts ET-1 into its active form, block DNA synthesis in glioblastoma cells [20]. ET-1 induces proliferation in glioblastoma through various pathways including the mitogen-activated protein kinase (MAPK) pathway, and BQ788, an ETRB-specific receptor antagonist, blocks the phosphorylation of extracellular signal-related kinase, a key step in MAPK signaling [21]. This led us to consider whether potential therapeutic candidates, the ETRB antagonists, negatively impact glioma growth.

Our laboratory previously showed that high levels of BQ788 inhibit melanoma proliferation both *in vitro *and *in vivo *[22]. We are currently investigating the effects of ETRB antagonists on melanoma and glioma, with particular interest in two ETRB-specific antagonists, BQ788, a peptide, and A-192621, an orally bioavailable small molecule. In the present work we demonstrate that both ETRB antagonists decrease the number of viable cells in melanoma and glioma cultures, while an ETRA-specific antagonist, BQ123, has no effect. In glioma cells, A-192621 induces cell cycle arrest, apoptosis and expression of DNA-damage associated genes. Surprisingly, however, the down-regulation of ETRB levels has no effect on the reduction in cell number by either ETRB antagonist.

## Methods

### Cells and cell culture conditions

The human glioma cell lines LN-229 and SW1088 and the human melanoma cell line A375 (American Type Culture Collection (ATCC), Manassas, VA, USA) were maintained in Dulbecco's Modification of Eagle's Medium (DMEM) (Mediatech, Inc., Herndon, VA, USA) and the human melanoma cell line WM35 (ATCC) was maintained in Eagle's Minimum Essential Medium (MEM) (Mediatech, Inc.). All cells were supplemented with 10% fetal bovine serum (FBS) (Gemini Biological Products, Calabasas, CA, USA), 100 units/mL penicillin and 100 μg/mL streptomycin (Invitrogen, Carlsbad, CA, USA) and cultured in a humidified incubator with 5% CO_2 _at 37°C. For cell viability assays, 2.5 × 10^4 ^cells were plated onto 12-well tissue-culture treated plates (Fisher Scientific, Pittsburgh, PA, USA) using media supplemented with 1% FBS. A-192621 (Abbott Laboratories, Abbott Park, IL, USA), BQ788 (EMD Chemicals Inc., San Diego, CA, USA) and/or BQ123 (EMD) were added 24 h after plating and viable cell number was assessed using the Live/Dead Viability/Cytotoxicity Kit for mammalian cells (Invitrogen) according to the manufacturer's instructions. Fluorescent intensity was measured on an FLx800 multi-detection microplate reader (BioTek, Winooski, VT, USA) and values represent the mean of a 25-point well scan.

### Cell proliferation and cell death

LN-229 and SW1088 cells were plated at 5 × 10^5 ^cells per 100 mm dish in DMEM with 1% FBS, and A-192621 was added 24 h later. Cell cycle analysis was performed with a BrdU/propidium iodide double stain using the Absolute-S Cell Proliferation Kit (eBioscience, San Diego, CA, USA) according to the manufacturer's instructions allowing 40 minutes to pulse-label cells with BrdU. Fluorescent intensity was measured using the BD FACSCalibur System (Becton Dickinson, Franklin Lakes, NJ, USA). The rate of cell proliferation was assessed using the CellTrace CFSE Cell Proliferation Kit (Invitrogen) according to the manufacturer's instructions. Cells were labeled with carboxyfluorescein diacetate succinimidyl ester (CFSE) at the time of plating and fluorescent intensity was analyzed at 9, 24, 48 and 72 h after the addition of A-192621 using the BD FACSCalibur System. Cell death was quantified by staining cells with propidium iodide (Invitrogen) following treatment with A-192621. Fluorescent intensity was analyzed using the BD FACSCalibur System. All FACS data was analyzed with FlowJo (Tree Star, Inc., Ashland, OR, USA). Caspase 3/7 activity was measured using EnzChek Caspase-3 Assay Kit #2 (Invitrogen) according to the manufacturer's instructions and values adjusted for total cell number. Fluorescent intensity was measured using the FLx800 multi-detection microplate reader.

### Microarray analysis

LN-229 and SW1088 cells were treated with vehicle, 10 nM or 100 μM A-192621 for 12 h and total RNA was prepared using the RNeasy Plus Mini Kit (Qiagen, Valencia, CA, USA). The quality of the samples was checked using the RNA 6000 Nano LabChip kit (Agilent Technologies, Santa Clara, CA, USA). RNA samples were then processed according to the Affymetrix Eukaryotic Sample and Array Processing protocol. Hybridization of the *in vitro *amplified RNA to Affymetrix Human Genome U133Plus 2.0 chips (Affymetrix, Inc., Santa Clara, CA, USA), washing and scanning of the arrays were performed following standard Affymetrix protocols using a Hybridization Oven 640, a Fluidics Station 450, and a GeneChip^® ^Scanner 3000 7G. The raw data (*.cel files) from the Affymetrix hybridizations were processed and analyzed using Resolver (Rosetta Biosoftware, Seattle, WA, USA). Genes were identified using cutoffs of fold-change > 3 and p < 0.0001.

### Real-time reverse-transcription PCR

Total RNA was prepared using the RNeasy Plus Mini Kit (Qiagen, Valencia, CA, USA). The cDNA was prepared from 1 μg total RNA using the Transcriptor First Strand cDNA Synthesis Kit (Roche Applied Science, Indianapolis, IN, USA) according to the manufacturers instructions. Primer/probe design was accomplished using the Universal ProbeLibrary (UPL) Assay Design Center (Roche Applied Science). The primer sets (Integrated DNA Technologies, Coralville, IA, USA) were as follows: 5'-GGC AGA AGC TGA AAG GTC TC-3' and 5'-CAT CGA AGC ACT GTC TCA GAG T-3' (DR5), 5'-GGA GAG CAG AAG ACC GAA AG-3' and 5'-AGT GAT CGT GCG CTG ACT C-3' (GADD45A), 5'-GCT TCT GGC AGA CCG AAC-3' and 5'-GTA GCC TGA TGG GGT GCT T-3' (GADD34), 5 '-ACT GCG TCT TTG GCA TCA G-3' and 5'-GTA GCA GGC CAC TGT CTT GA-3' (Sestrin 2), 5'-AAG GCA CTG AGC GTA TCA TGT-3' and 5'-TGA AGA TAC ACT TCC TTC TTG AAC AC-3' (GADD153), 5'-TTC ATC CCG TTC AGA AGA CA-3' and 5'-CCA ATG GCA AGC AGA AAT AGA-3' (ETRB) and 5'-TGA CCT TGA TTT ATT TTG CAT ACC-3' and 5'-CGA GCA AGA CGT TCA GTC CT-3' (HPRT). The corresponding probes were UPL probe #63 (DR5), #65 (GADD45A), #28 (GADD34), #17 (Sestrin 2), #21 (GADD153), #83 (ETRB) and #73 (HPRT) (Roche Applied Science). PCR was performed and analyzed on a LightCycler 480 System (Roche Applied Science) using LightCycler 480 Probes Master (Roche Applied Science). The PCR was done under the following conditions: pre-incubation at 95°C for 5 minutes, 45 cycles of amplification with melting at 95°C for 8 seconds, annealing at 60°C for 15 seconds and extension at 72°C for 2 seconds, and 1 cycle of cooling at 40°C for 10 seconds. All gene expression was quantified relative to HPRT expression.

### Small interfering RNA (siRNA)

Following the reverse transfection protocol, Lipofectamine RNAiMAX (Invitrogen) was diluted in Opti-MEM I Medium (Invitrogen) and ON-TARGETplus SMARTpool ETRB siRNA or ON-TARGETplus siCONTROL non-targeting siRNA (Dharmacon, Lafayette, CO, USA) was diluted in Opti-MEM I Medium without serum. These solutions were then combined and incubated together at room temperature for 10–20 minutes. The siRNA duplex-Lipofectamine RNAiMAX complexes were then plated and overlaid with 2.5 × 10^4 ^cells per ml in media with 1% FBS. Media was changed 4–6 h after plating and cells were treated as described above. ETRB gene expression was assessed by real-time PCR and was reduced by 70 – 93%.

### Statistical analysis

Values are expressed as mean ± SEM. Statistical analysis was done using one-way ANOVA with Tukey posthoc unless otherwise noted. P < 0.05 was considered statistically significant.

## Results

### Endothelin receptor B antagonist A-192621 reduces the number of viable glioma and melanoma cells in a dose- and time-dependent manner

**Figure 1:**
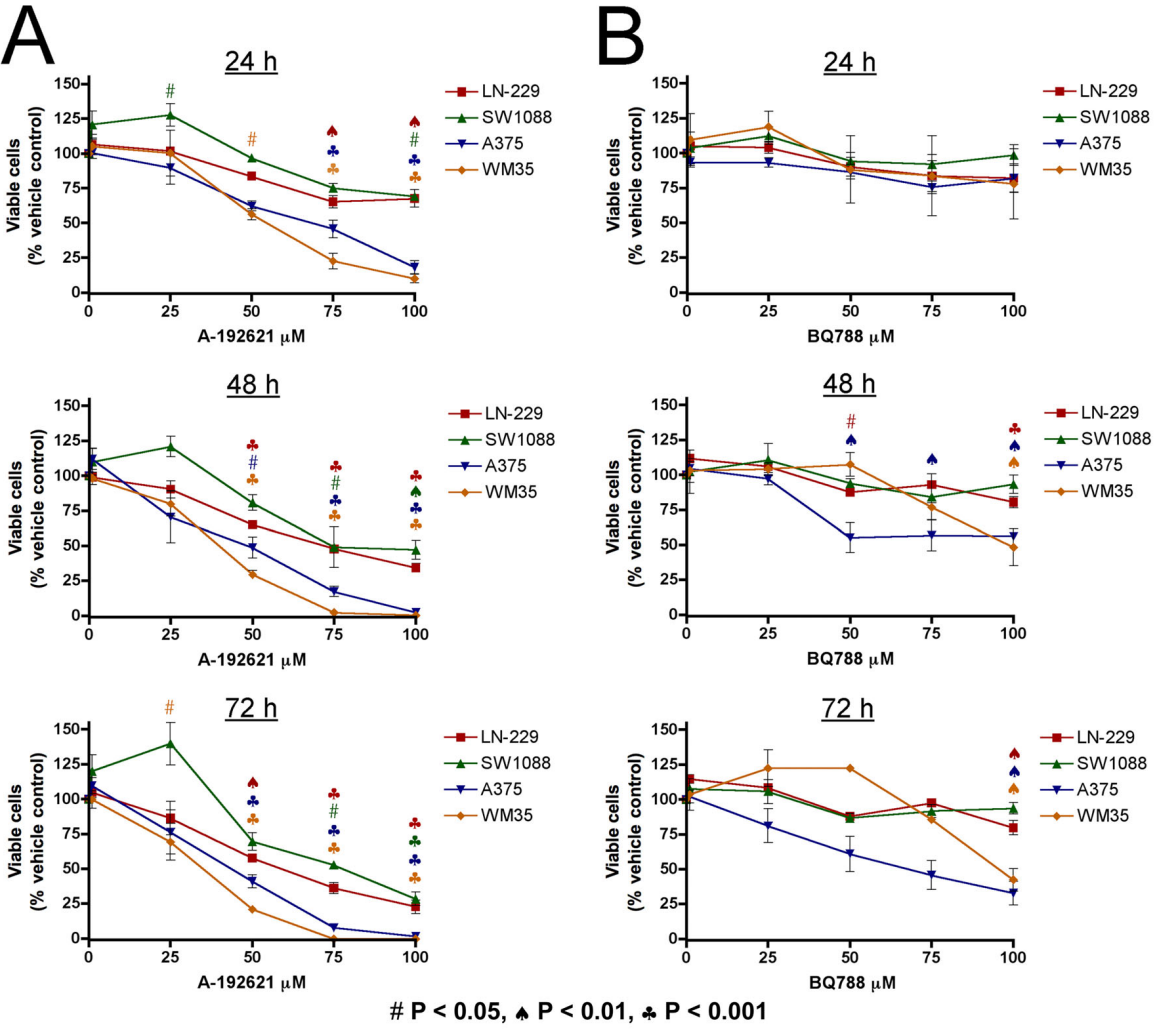
**Endothelin receptor B antagonists reduce viable cell number in a dose- and time-dependent manner**. (A) A-192621 significantly reduces the number of viable cells in both glioma (LN-229 and SW1088) and melanoma (A375 and WM35) cell lines at 24, 48 and 72 h of treatment. (B) BQ788 significantly reduces the number of viable cells in melanoma at 48 and 72 h of treatment. Values are expressed as the mean of three replicates ± SEM. Symbols for statistical significance as compared with vehicle-treated controls are displayed at the bottom of the figure and are applicable to all panels. Colors correspond to the cell line.

To test the effectiveness of A-192621 in reducing viable glioma cells we employed two human glioma cell lines, LN-229 and SW1088. LN-229 was originally derived from a grade IV glioblastoma and SW1088 was derived from an anaplastic astrocytoma. Twenty-four hours after plating, A-192621 was added at concentrations from 1 to 100 μM to non-confluent cells and incubated for 24 to 72 h. Viable cells were quantified by the capacity of their intracellular esterases to convert non-fluorescent calcein AM into green-fluorescent calcein. This assay reveals a decrease in viable cells with increasing A-192621 concentration (Fig. 1A). This decrease is enhanced at longer incubation times (Fig. 1A). In addition, A-192621 is effective in reducing viable cells in the human melanoma cell lines A375 and WM35 in a dose- and time-dependent manner (Fig. 1A). This finding with melanoma cells is consistent with previously published data reporting that A-192621 inhibits melanoma growth in nude mice [13]. We also tested the ability of ETRB-specific antagonist BQ788 to reduce the viability of the glioma cells. BQ788 was added to non-confluent cells in the same manner as A-192621 at concentrations from 1 to 100 μM. This inhibitor causes a significant decrease in viable LN-229 cells after 48 or 72 h of treatment but no significant change in SW1088 cells (Fig. 1B). BQ788 also reduces cell viability in both melanoma cell lines in a dose- and time-dependent manner (Fig. 1B). This last result is consistent with previous findings of melanoma cells in our laboratory [22]. To assess the involvement of ETRA in viability, cells were treated with BQ123, an ETRA-specific antagonist. BQ123 was added to cells at 1 to 100 μM and incubated for 24 to 72 h. No significant changes in cell viability were observed in any of the four cell lines, at any concentration or time point (Additional file 1). Thus, potential cross-reaction of A-192621 or BQ788 with ETRA does not play a role in their effects on cell viability.

### A-192621 decreases glioma cell proliferation and increases cell death

**Figure 2:**
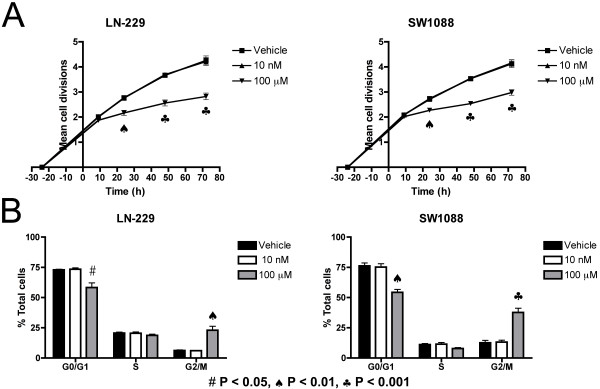
**The endothelin B receptor antagonist A-192621 decreases glioma cell proliferation**. (A) A-192621 suppresses cell proliferation. LN-229 and SW1088 cells were stained with CFSE and analyzed by FACS at 9, 24, 48 and 72 h after addition of A-192621. The exponential dilution of CFSE was converted to the number of cell divisions. (B) A high concentration of A-192621 (100 μM) induces G2/M cell cycle arrest in both LN-229 and SW1088 cells by 72 h. Cells were pulsed-labeled with BrdU, stained with propidium iodide and analyzed by FACS. Values are expressed as means of three replicates ± SEM. Symbols for statistical significance as compared with vehicle-treated controls are displayed at the bottom of the figure and are applicable to all panels.

To investigate how A-192621 reduces glioma cell number we assessed the rate of cell proliferation over time. The human glioma cells were labeled with carboxyfluorescein diacetate succinimidyl ester (CFSE). This non-fluorescent reagent passively diffuses into cells, where the acetate groups are cleaved by intracellular esterases, producing green-fluorescent carboxyfluorescein succinimidyl ester. The ester groups react with intracellular amines and fluorescent intensity is exponentially diluted as cells divide. Fluorescent intensity was measured by FACS at 9 to 72 h. We tested two doses of A-192621, 10 nM, a concentration slightly above the IC_50 _that is calculated from radio-labeled ET-1 binding displacement studies [23], and 100 μM, a concentration that dramatically reduces the number of viable cells in both the LN-229 and SW1088 lines (Fig. 1A). We find that 100 μM, but not 10 nM, significantly reduces fluorescent carboxyfluorescein succinimidyl ester dilution in both LN-229 and SW1088 cells at 24 h, and this effect is sustained through later time points, indicating that A-192621 reduces the rate of cell division (Fig. 2A).

**Figure 3:**
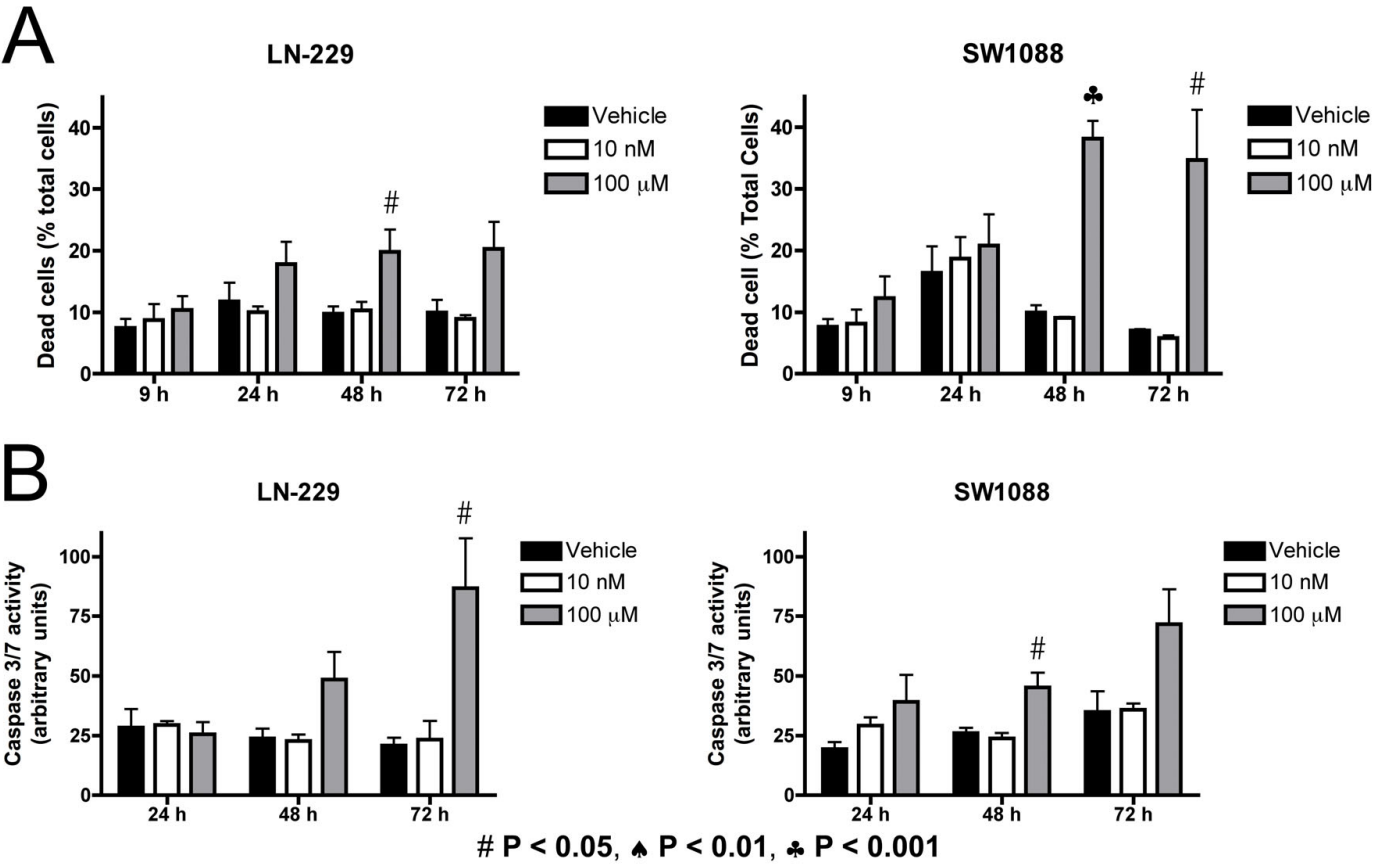
**The endothelin B receptor antagonist A-192621 induces cell death**. (A) A-192621 induces cell death at 48 and 72 h. LN-229 and SW1088 cells were stained with propidium iodide following treatment with A-192621 and analyzed by FACS. (B) A-192621 induces apoptosis by 72 and 48 h in LN-229 and SW1088 cell lines respectively. Caspase 3/7 activity was adjusted for total cell number. Values are expressed as means of three replicates ± SEM. Symbols for statistical significance as compared with vehicle-treated controls are displayed at the bottom of the figure and are applicable to all panels.

We also examined the cell cycle status following A-192621 treatment using BrdU/propidium iodide (PI) double staining. Following treatment with A-192621, cultures were labeled with BrdU for 40 min to identify cells undergoing DNA synthesis and fixed immediately afterwards. Cells were analyzed by FACS, and BrdU intensity was plotted against DNA content. In both glioma cell lines, 100 μM A-192621 significantly increases the percentage of cells in the G2/M phase compared to vehicle, or to 10 nM treatment after 24 h (data not shown). This accumulation of cells in the G2/M phase continues through 72 h and is coupled with a concomitant decrease in the G1/G0 population (Fig. 2B), indicating that A-192621 induces a G2/M phase arrest. In addition to effects on proliferation, we investigated whether A-192621 treatment also affects cell death, as measured by the percentage of total cells that stain positively with PI. A-192621 significantly increases PI staining at 48 h in both LN-229 and SW1088 cells, and at 72 h in SW1088 cells (Fig. 3A). Moreover, as a measure of apoptotic cell death, caspase 3/7 activity is increased at 72 and 48 h in LN-229 and SW1088 cells, respectively (Fig 3B).

### Genes induced by DNA damage are up-regulated following A-192621 treatment

In order to further understand the effects of A-192621 on glioma cells, we assessed changes in gene expression by microarray analysis following a 12 h treatment with vehicle, 10 nM or 100 μM A-192621 in glioma cells. Changes in gene expression were identified using the criteria of > 3-fold change and P < 0.0001. A striking finding is that, in both cell lines, 100 μM A-192621 up-regulates several genes that are known to be induced by DNA damage (Table 1). These genes were not significantly up-regulated by 10 nM A-192621. These genes include growth arrest and DNA-damage-inducible (GADD) 153, GADD45A, GADD34, sestrin 2 and death receptor 5 (DR5). The up-regulation of these genes was confirmed by real-time PCR in the human glioma cell lines, and also in the human melanoma cell lines (Table 1). A-192621 increases GADD153 expression the most dramatically, with a 10- to 66-fold increase, depending on the cell line treated. A-192621 increases GADD45A and GADD34 expression similarly by 5- to 23- and 3- to 21-fold, respectively. Expression of sestrin 2 and DR5 is up-regulated 12- to 19- and 4- to 12-fold, respectively.

**Table 1:** Treatment of glioma and melanoma cell lines with 100 μM A-192621 up-regulates DNA damage-inducible genes.

**Genes**	**Cell Lines**			
	*LN-229*	*SW1088*	*A375*	*WM35*
GADD153	28	12	10	66
GADD45A	23	5	9	8
GADD34	16	3	21	6
Sestrin 2	15	18	19	12
DR5	12	4	10	5

Genes were identified by microarray analysis and real-time RT-PCR was used to quantify the fold-increase in expression over vehicle-treated controls.

### A-192621 and BQ788 effects on cell viability *in vitro *are not mediated by endothelin receptor B

**Figure 4:**
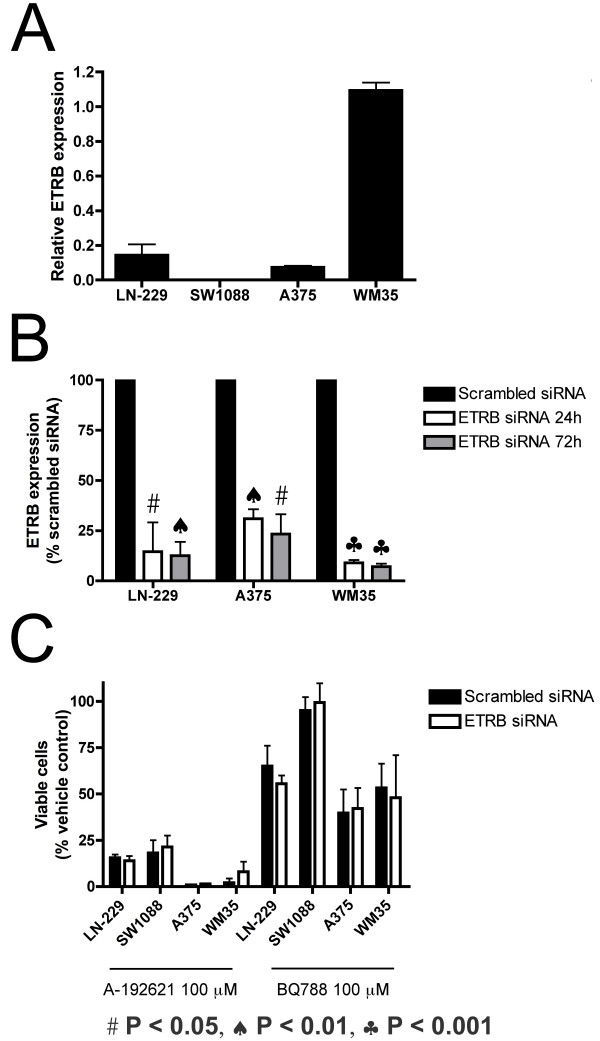
**Reduced ETRB expression does not alter the effect of ETRB antagonists on viable cell number**. (A) ETRB mRNA in untreated cell lines. ETRB mRNA was not detected in the SW1088 line. (B) Following transfection, reduction in ETRB mRNA was assessed by real-time RT-PCR. Reduction in ETRB expression is displayed as a percentage relative to ETRB mRNA in cells transfected with scrambled siRNA. (C) Cells were transfected with either scrambled siRNA or ETRB siRNA and then treated with either 100 μM A-192621 or 100 μM BQ788 for 72 h. Values are expressed as a mean of three replicates ± SEM. Significance was determined by a paired t-test. Symbols for statistical significance are displayed at the bottom of the figure and are applicable to panels B and C.

The concentrations A-192621 and BQ788 required to reduce cell viability *in vitro *are far above the IC_50 _concentrations required to displace ET-1 [23,24]. Moreover, when we examined expression levels by real-time PCR, we could not detect ETRB in the SW1088 cell line after 45 amplification cycles (Fig 4A). These findings led us to question the involvement of ETRB in the growth inhibition by ETRB antagonists. To address this question directly, we reduced the expression level of ETRB 69–93%, using small interfering RNA (siRNA) (Fig 4B). All cell lines were transfected with siRNAs targeting ETRB or a non-targeting, scrambled siRNA, and then treated 24 h later with 100 μM A-192621, BQ788 or their respective vehicles. The number of viable cells was assessed at 24 to 72 h. First, reduced ETRB expression alone does not affect cell viability. Second, both A-192621 and BQ788 decrease the number of viable cells equivalently in cells transfected with ETRB-targeting siRNA or scrambled siRNA at all time points (Fig. 4C; 24 and 48 hours not shown). The lack of effect of ETRB knockdown on A-192621- and BQ788-mediated reduction in cell number is further evidence that antagonism of this receptor is not the primary mechanism for the viability effects of these two drugs *in vitro*.

## Discussion

A number of ETRB antagonists are widely used in studies of cancer, and several have been tested in humans. We show here that both melanoma and glioma cell viability are sensitive to ETRB antagonists. Both BQ788 and A-192621 decrease melanoma and glioma cell number in a dose- and time-dependent manner. We find that A-192621 is more potent than BQ788, causing a greater decrease in viable cell numbers at lower concentrations and at earlier time points. In fact, within the time frame tested, only A-192621 was able to reduce the viable cell number in the astrocytoma line SW1088.

In addition, A-192621 is attractive as a therapeutic agent because it is orally bioavailable, and considering that it is also more potent than BQ788, we investigated how it reduces glioma cell number in greater detail. The CFSE labeling study indicates that A-192621 inhibits mitosis. Using cell cycle analysis, treatment of glioma cells with A-192621 increases the percentage of cells with G2/M DNA content over time. This is coupled with a concomitant decrease in the percentage of cells with G0/G1 DNA content, indicating an arrest in the G2/M phase of the cell cycle, which likely accounts for the reduction in cell division seen with CFSE labeling. We also find that A-192621 treatment induces apoptotic cell death. Reduction in viable cell number is therefore a consequence of both decreased mitosis and increased apoptosis. To our knowledge, this is the first evidence of ETRB antagonist-induced G2/M cell cycle arrest.

To further elucidate the actions of A-192621 on human glioma cells, we analyzed changes in gene expression using microarray technology. Notably, after 12 h of A-192621 treatment, there are highly significant increases in the expression of several genes known to be up-regulated following DNA damage. These genes include GADD153, GADD45A, GADD34, Sestrin 2 and DR5. Environmental stressors such as methylmethane sulfonate, or ultraviolet or gamma irradiation induce GADD45A [25]. Expression of GADD45A and other GADD45-like genes activates the p38/JNK pathway and apoptosis. GADD45A also induces G2/M cell cycle arrest through its interaction with Cdc2 and cyclin B1 following genotoxic stress [26-29]. Two other members of the GADD family, GADD34 and GADD153, are also up-regulated by A-192621 treatment. Like other members of this family, GADD34 and GADD153 are induced by stressful growth conditions and DNA damage. Over-expression of GADD34 and GADD153, along with GADD45 and others, suppresses cell growth (as measured by colony formation) and induces apoptosis [30-33]. Sestrin 2 is one of three closely related genes in the sestrin family [34] and is closely linked to other GADD genes since sestrin 1, also known as PA26 [35], is a member of the GADD gene family. Sestrin 2, also known as Hi95, is induced by hypoxia, oxidative stress and DNA damage [36]. Over-expression of sestrin 2 leads to apoptosis approximately 24 h following induction, and the cells are hypersensitive to further insult. DR5 is one of the TRAIL receptors with a cytoplasmic death domain that induces caspase-dependent apoptosis [37-39]. DR5 is induced by DNA damaging compounds in malignant gliomas, including LN-229 [40]. Taken together, this evidence suggests that A-192621 affects glioma viability by activating stress/DNA damage response pathways, which leads to cell cycle arrest and apoptosis. A similar process may also occur in melanoma. The up-regulation of these genes was confirmed by real-time PCR in LN-229 and SW1088 cell lines, and also occurs in human melanoma cell lines, A375 and WM35, following 12 h of A-192621 treatment. Up-regulation of these genes may account for the G2/M arrest and the apoptosis we see at later time points. This is the first evidence linking ETRB antagonist treatment to enhanced expression of DNA damage-inducible genes.

We also present evidence that the reduction of both glioma and melanoma *in vitro *viability by A-192621 and BQ788 is not dependent on ETRB expression. This conclusion is supported by three types of data. (1) The concentrations of ETRB antagonists required to reduce cell number are far above the concentrations required to displace ET-1 from ETRB. (2) At the high dose, A-192621 reduces cell viability in the glioma cell line SW1088 despite the absence of detectable ETRB expression in these cells. (3) Experimental reduction of ETRB expression in the other cell lines by >90% has no effect on the ability of either antagonist to reduce glioma or melanoma cell numbers *in vitro*. Despite the evidence that these ETRB antagonists are not acting through ETRB, it is clear that they are not toxic for all cell types. That is, our prior experiments showed that BQ788 kills 7 different melanoma cell lines without affecting the human kidney line 293, even at 100 μM [23]. Thus, there appears to be something distinctive about melanoma and glioma cells, and possibly a number of cancer cell types (ovarian, prostate, meninges) that are susceptible to ETRB antagonists [2].

## Conclusion

We have demonstrated that ETRB antagonists are effective agents against glioma and melanoma cell growth *in vitro*. To date, mechanisms of ETRB antagonist action in cancer treatment have focused on blocking ET-1 induced pathways. Although determining the precise mechanism by which ETRB antagonists reduce cell number in these cancers is beyond the scope of this study, the data presented here indicate that ETRB antagonists function independently of direct ETRB antagonism to mediate their effects on *in vitro *cell viability. We present evidence that A-192621 affects glioma and melanoma viability by activating stress/DNA damage response pathways, which leads to cell cycle arrest and apoptosis. This is the first evidence linking ETRB antagonist treatment to enhanced expression of DNA damage-inducible genes, and suggests a novel direction for future work on the mechanism of action of ETRB antagonists in cancer.

## Competing interests

The authors declare that they have no competing interests.

## Authors' contributions

JPM participated in the design of the study, drafted the manuscript and carried out the experimental work. PHP participated in the study conception, coordination and manuscript preparation. Both authors read and approved the final manuscript.

## Pre-publication history

The pre-publication history for this paper can be accessed here:



## Supplementary Material

Additional file 1 **The endothelin receptor A-specific antagonist BQ123 does not affect viable cell number in glioma or melanoma cell lines**. Cells were treated with BQ123 for 72 h. Values are expressed as means of three replicates ± SEM. Symbols for statistical significance as compared with vehicle-treated controls are displayed at the bottom of the figure and are applicable to all panels. Click here for file
