# Supplementary figures and images for: Endothelin receptor B antagonists decrease glioma cell viability independently of their cognate receptor
Source: BMC Cancer. 2008 Nov 28;8:354. doi: 10.1186/1471-2407-8-354 (PMC2613414; doi:10.1186/1471-2407-8-354)

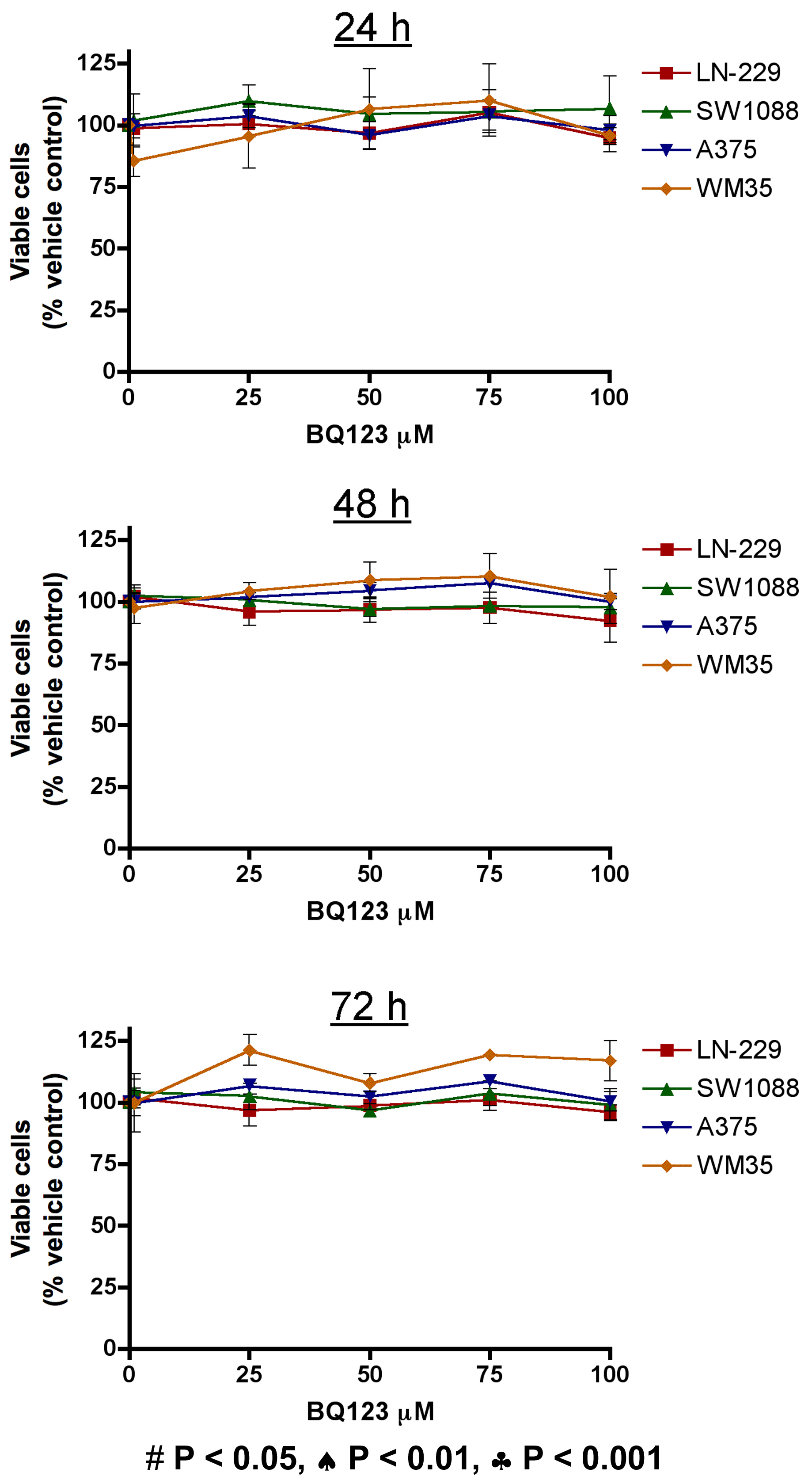

Supplement: Additional file 1 — The endothelin receptor A-specific antagonist BQ123 does not affect viable cell number in glioma or melanoma cell lines. Cells were treated with BQ123 for 72 h. Values are expressed as means of three replicates ± SEM. Symbols for statistical significance as compared with vehicle-treated controls are displayed at the bottom of the figure and are applicable to all panels. [file 1471-2407-8-354-S1.jpeg]
